# Immune checkpoint inhibitors and response analysis: a tough challenge. A case report

**DOI:** 10.1186/s13104-016-2153-9

**Published:** 2016-07-18

**Authors:** Alessandra Bearz, Tiziana Perin, Luca Cancian, Eleonora Berto, Ivana Sartor, Umberto Tirelli

**Affiliations:** Division of Medical Oncology A, Department of Medical Oncology, Centro di Riferimento Oncologico, National Cancer Institute of Aviano, Via Franco Gallini 2, 33081 Aviano, PN Italy; Division of Pathology, Centro di Riferimento Oncologico, National Cancer Institute, Aviano, PN Italy; Division of Radiology, Centro di Riferimento Oncologico, National Cancer Institute, Aviano, PN Italy; Clinical Trial Office, Scientific Directorate, Centro di Riferimento Oncologico, National Cancer Institute, Aviano, PN Italy

**Keywords:** NSCLC, Immune-checkpoint inhibitors, Radiologic response

## Abstract

**Background:**

Treatment of metastatic NSCLC patients with immune-checkpoint medicine is intriguing for the potential efficacy; however it may be difficult to evaluate the clinical response due to the lack of reliable immune-monitoring markers up to now and the possibility of radiological pseudo-progression.

**Case presentation:**

Herein we report the case of a patient ex-smoker with adenocarcinoma of the lung, stage IV for liver metastases, in progression after cisplatin-based chemotherapy and treated with antiPD-L1 (MPDL3802-Roche Genentech) e.v. every 3 weeks in a clinical trial. Treatment with antiPD-L1 was well tolerated and CT scan after 6 weeks of treatment showed stabilization of mediastinal lymph nodes, while progression of liver metastases; liver progression only was confirmed by further CT-scans. Patient was asymptomatic and it was unclear if we faced a pseudo-progression in the liver or a real progression. Data about his PDL1 expression were not available because the patient was in a clinical trial. Eventually a biopsy of the liver metastasis confirmed that there was a massive neoplastic invasion with tumor infiltrating lymphocytes <5 %. We stopped anti-PD-L1 therapy due to progression.

**Conclusion:**

Evaluation of response may be difficult with immune checkpoint inhibitors, in particular radiologic images may be a matter of debate; eventually we performed a biopsy to study tumor infiltrating lymphocytes to decide whether it was pseudo-progression or real progression.

## Background

Response assessment during anticancer treatment is a strategic checkpoint to decide whether keep the patient on treatment or stop it and eventually change it. According to the RECIST system, dimensional criteria have driven such decision up to now; however, immune-checkpoint inhibitors have started a paradigm shift. There may be a dimensional increase of the tumor, without clinical deterioration and we do not know if refer it as pseudo-progression or real progression [1]. Hodi et al. described two types of pseudo-tumor progression in melanoma, early and delayed, according to the timing of presentation [2], meaning an initial progression followed by a further shrinkage of the neoplastic disease, without changing of treatment. Although it is not a frequent phenomenon (the incidence of response with distinct immune-related patterns of response across several solid tumors is roughly 4 %), it is extremely important to recognize it avoiding inappropriate discontinuation of therapy [3, 4].

Response evaluation on immune-checkpoint inhibitors is a new and challenging issue for oncologists; although response rate is not the ultimate goal, it is a key tool to decide whether to keep the patient on treatment. Therefore we present our case, where the progression of disease was unclear at the beginning and a histological confirmation was necessary to understand the efficacy of the treatment.

## Case presentation

**Fig. 1:**
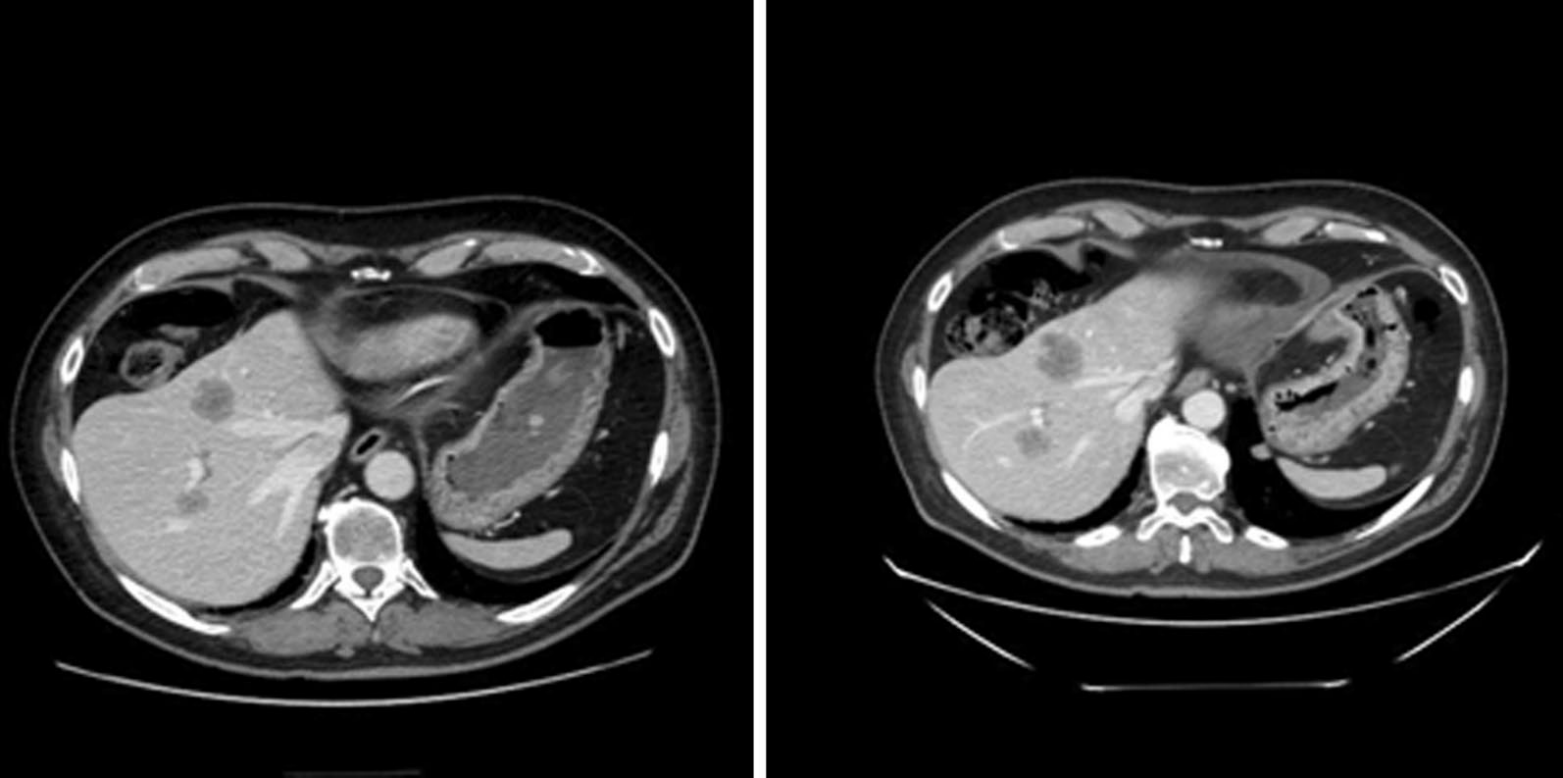
Metastatic localizations in the liver (VII and VIII segment) in the CT-scan of September 2014 (on the *left*), before treatment with antiPD-L1, and November 2014, after 6 weeks of treatment with antiPD-L1 (on the *right*) (H&E, ×20). The *arrow* shows the metastatic localization where the biopsy was performed

A 61 year-old, male patient arrived at our Institute in September 2014, with an adenocarcinoma of the lung, EGFR wild-type and ALK not-translocated, involving the mediastinum and the liver where there were three little metastatic localizations. His performance status (PS) score was 0, he was asymptomatic and his hepatic and renal functions were normal. He had already received 4 cycles of cisplatin combined with pemetrexed, obtaining stable disease; he was then put on maintenance treatment with pemetrexed and eventually after 5 cycles he developed progression at the liver. The patient had been referred to us for entering a clinical trial. We enrolled him into a protocol with anti PD-L1 (MPDL3802-Roche Genentech), 10 mg/kg i.v. every 3 weeks. His PD-L1 status was not disclosed, being in a clinical trial. After 6 weeks the computed tomography (CT)-scan demonstrated progression of all the hepatic lesions (Fig. 1), while the mediastinum remained stable; after 12 weeks CT-scan again showed progression at the liver, 18 weeks later radiologic evaluation demonstrated once more progression of the three liver nodules and progression of the mediastinal lymph nodes. No new lesions were observed. Liver function remained normal and his PS was 0.

To understand the underlying process, whether it was pseudo-progression or real progression, after 12 weeks of treatment with anti-PD-L1 we performed two biopsies of one liver metastasis; we chose to biopsy the liver localization, because it was easy to reach and in progression from the very beginning of treatment.

## Methods

Surgical specimens were sampled according to current protocols. Formalin-fixed, paraffin-embedded tissue samples were obtained, 4-μm sections were stained with hematoxylin and eosin 2.5-μm sections were cut and immunohistochemical analysis was performed in an automated system (Benchmark-XT, Ventana, Tucson, AZ, US). The following primary antibodies were used: TTF-1 (monoclonal antibody, clone SP141, pre-diluted; Ventana, Tucson, AZ, US), CD45 (monoclonal antibody, clone 2B11&PD7/26; prediluted; Ventana, Tucson, AZ, US) and CD3 (monoclonal antibody, clone 2GV6; Ventana, Tucson, AZ, US). Color was developed with 3.3′-diaminobenzidine (DAB) and slides were counterstained with Meyer’s hematoxylin. Appropriate positive and negative controls were concurrently done.

## Conclusions

**Fig. 2:**
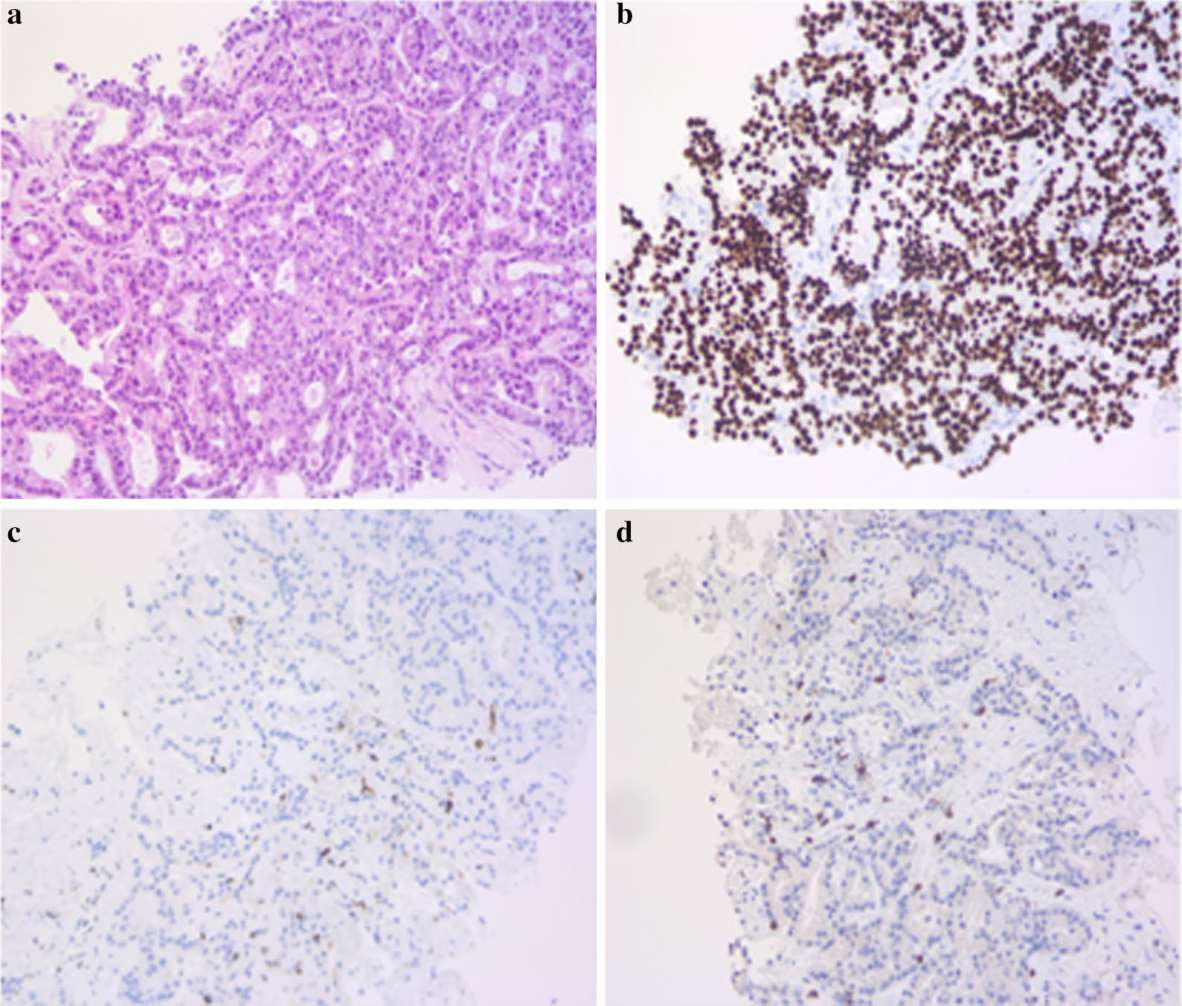
Histologic features of the metastatic infiltrate in the liver (**a**), and immunohistochemistry for TTF-1 (**b**), CD45 (**c**) and CD3 (**d**) (H&E, ×20)

We analyzed the percentage of lymphocyte infiltration versus the cancer burden, overall we found less than 5 % of lymphocytes (Fig. 2). There is no robust existing literature about the typical percentage of lymphocytes infiltrating a tumor as a sign of immune- response against the tumor; there is one report about a case of melanoma, where a cutaneous leg lesion obtained enlargement during the early phases of treatment with ipilimumab and was excised because of bleeding; histopathology of the lesion showed a high proportion of infiltrating T lymphocytes, roughly more than 30 %, while the outcome of the patient turned out to be positive, with a long-lasting stability for more than 20 months [5].

Since we did not find any dense infiltrate of lymphocytes in the liver biopsies, we concluded that our patient had a real progression and stopped the treatment with anti PD-L1.

Up to now there are no available and reliable predictive factors for immune-checkpoint inhibitors neither dynamic predictive markers of efficacy; the tumoral response may be difficult to assess for the pseudo-progression phenomena [3].

Until a reliable clinical or biological predictor marker of activity for this new class of anticancer drugs is available and until radiological evaluation of response is based on dimension of cancer nodules, the analysis of response could be a real challenge in patients on treatment with immune-checkpoint inhibitors. In our case, the presence of an easily percutaneously accessible metastasis allowed a bioptic assessment to understand the real efficacy of the ongoing treatment.

## Abbreviations

PS: performance status
NSCLC: non-small cell lung cancer
EGFR: epidermal growth factor receptor
ALK: anaplastic lymphoma kinase
TTF-1: thyroid transcription factor 1
CT-scan: computed tomography scan
RECIST: response evaluation criteria in solid tumors
